# Perceptions on Barriers and Facilitators to Colonoscopy Completion After Abnormal Fecal Immunochemical Test Results in a Safety Net System

**DOI:** 10.1001/jamanetworkopen.2021.20159

**Published:** 2021-08-10

**Authors:** Rachel B. Issaka, Ari Bell-Brown, Cyndy Snyder, Dana L. Atkins, Lisa Chew, Bryan J. Weiner, Lisa Strate, John M. Inadomi, Scott D. Ramsey

**Affiliations:** 1 Clinical Research Division, Fred Hutchinson Cancer Research Center, Seattle, Washington; 2 Hutchinson Institute for Cancer Outcomes Research, Fred Hutchinson Cancer Research Center, Seattle, Washington; 3 Division of Gastroenterology, University of Washington School of Medicine, Seattle; 4 Department of Family Medicine, University of Washington School of Medicine, Seattle; 5 Department of General Internal Medicine, University of Washington School of Medicine, Seattle; 6 Department of Health Services, University of Washington School of Public Health, Seattle; 7 Department of Internal Medicine, University of Utah School of Medicine, Salt Lake City

## Abstract

**Importance:**

The effectiveness of stool-based colorectal cancer (CRC) screening, including fecal immunochemical tests (FITs), relies on colonoscopy completion among patients with abnormal results, but in safety net systems and federally qualified health centers, in which FIT is frequently used, colonoscopy completion within 1 year of an abnormal result rarely exceeds 50%. Clinician-identified factors in follow-up of abnormal FIT results are understudied and could lead to more effective interventions to address this issue.

**Objective:**

To describe clinician-identified barriers and facilitators to colonoscopy completion among patients with abnormal FIT results in a safety net health care system.

**Design, Setting, and Participants:**

This qualitative study was conducted using semistructured key informant interviews with primary care physicians (PCPs) and staff members in a large safety net health care system in Washington state. Eligible clinicians were recruited through all-staff meetings and clinic medical directors. Interviews were conducted from February to December 2020 through face-to-face interactions or digital meeting platforms. Interview transcripts were analyzed deductively and inductively using a content analysis approach. Data were analyzed from September through December 2020.

**Main Outcomes and Measures:**

Barriers and facilitators to colonoscopy completion after an abnormal FIT result were identified by PCPs and staff members.

**Results:**

Among 21 participants, there were 10 PCPs and 11 staff members; 20 participants provided demographic information. The median (interquartile range) age was 38.5 (33.0-51.5) years, 17 (85.0%) were women, and 9 participants (45.0%) spent more than 75% of their working time engaging in patient care. All participants identified social determinants of health, organizational factors, and patient cognitive factors as barriers to colonoscopy completion. Participants suggested that existing resources that addressed these factors facilitated colonoscopy completion but were insufficient to meet national follow-up colonoscopy goals.

**Conclusions and Relevance:**

In this qualitative study, responses of interviewed PCPs and staff members suggested that the barriers to colonoscopy completion in a safety net health system may be modifiable. These findings suggest that interventions to improve follow-up of abnormal FIT results should be informed by clinician-identified factors to address multilevel challenges to colonoscopy completion.

## Key Points

**Question:**

What are the clinician-identified barriers and facilitators to colonoscopy completion among patients with abnormal fecal immunochemical test (FIT) results in a safety net health care system?

**Findings:**

In this qualitative study of 21 primary care physicians (PCPs) and staff members, barriers to follow-up colonoscopy completion included environmental factors (ie, social determinants of health and organizational factors) and patient cognitive factors. Resources that addressed these barriers (eg, insurance assistance, appointment reminders, and bowel preparation education) were associated with improved colonoscopy completion.

**Meaning:**

These findings suggest that interventions to improve colonoscopy completion among patients with abnormal FIT results should be informed by clinician-identified barriers and facilitators.

## Teaser

This qualitative study investigates clinician-identified barriers and facilitators to colonoscopy completion among patients in a safety net health care system who receive abnormal fecal immunochemical test (FIT) results.

## Introduction

There is clear evidence that screening for colorectal cancer (CRC) by stool-based tests is cost-effective^1^ and saves lives^2^; however, screening remains underused, especially among members of racial/ethnic minority groups and low-income populations.^3^ In safety net health care settings and federally qualified health centers (FQHCs) (eTable in the Supplement), in which many medically underserved populations receive care, CRC screening improves when a fecal immunochemical test (FIT) is offered alongside colonoscopy.^4^ Additionally, owing to patient preference and limited resources,^5^ FIT has become a cornerstone for CRC screening in these settings. Among patients with an abnormal FIT result, the estimated CRC prevalence is 3.4%^6^ and a missed or delayed diagnostic colonoscopy is associated with increased CRC mortality.^7,8^ Despite these concerns, the proportion of patients with an abnormal FIT result who complete a diagnostic colonoscopy rarely exceeds 50% in most safety net systems and FQHCs.^9,10,11^

At Harborview Medical Center (HMC), a safety net health care system for the Seattle region, among 299 adults ages 50 to 75 with an abnormal FIT result for CRC screening from 2014 to 2018, 122 individuals (40.8%), completed a colonoscopy within 1 year of their abnormal result (patient electronic health record [EHR] data obtained by R.B.I. from University of Washington Medicine Information Technology Services on November 20, 2019). Our prior work^10^ examining colonoscopy completion in a different safety net health care system similarly found inadequate colonoscopy completion rates. Colonoscopy completion is a complex process that requires effective communication and coordination among patients, primary and specialty care clinicians, and the health care system.^12^ In 2 studies from 2021, 31%^13^ and 46%^14^ of patients completed a colonoscopy within 6 months of their abnormal FIT results after receiving patient navigation. While these results are promising, they suggest the need for additional interventions to achieve the US Multi-Society Task Force (USMSTF) follow-up colonoscopy goal of 80%.

Improving follow-up colonoscopy completion is a priority for HMC leaders and administrators, but evidence-based guidance on where to intervene along the screening continuum is needed. A 2017 review^15^ of interventions to improve colonoscopy completion after abnormal stool-based CRC screening test results lacked representation from FQHCs or safety net health systems. A 2018 meta-analysis^16^ could not determine the overall effectiveness of any intervention because of the low number of available studies. Qualitative studies could fill an important knowledge gap and aid in the development of interventions to reach the 80% follow-up goal. There is also a need for studies that include safety net health systems and FQHCs. In these settings, understanding clinician-specific factors associated with colonoscopy completion may lead to more effective interventions to address this persistent issue. The aim of this study is to describe clinician-identified barriers and facilitators to colonoscopy completion among patients with abnormal FIT results. Our goal is to inform interventions aimed at improving follow-up of abnormal FIT results and CRC outcomes in FQHCs and safety net systems.

## Methods

In this qualitative study, we conducted semistructured, key informant interviews of primary care physicians (PCPs) and staff members within HMC from February to December 2020. This study adhered to the Consolidated Criteria for Reporting Qualitative Research (COREQ) reporting guideline^17^ and was approved by the Fred Hutch/University of Washington Cancer Consortium’s institutional review board. All participants provided verbal consent to be recorded and to have their data and responses published. Each participant received a $100 cash incentive for their time.

### Study Setting and Population

HMC is a safety net county teaching hospital system in Seattle, Washington, with 7 primary care clinics that provide care to historically underserved populations in King County, including individuals of lower socioeconomic status, who are uninsured, and whose primary language is not English. All HMC clinics, through their affiliation with the University of Washington, share a single integrated EHR.

### Sampling and Recruitment

Clinic medical directors identified HMC PCPs and staff members involved in follow-up of abnormal FIT results. We then recruited a convenience sample of participants from the 7 HMC primary care clinics through all-staff meetings. Study staff contacted interested individuals by email for further eligibility assessment and enrollment. Participants were eligible if they were employed by an HMC primary care clinic that provided care to adults aged 18 years and older and personally provided care to adults aged 50 years or older who used FIT for CRC screening. Participants included PCPs (ie, attending physicians and residents) and staff members (ie, physician assistants, nurses, patient care coordinators, medical assistants, clinic managers, and caseworkers). By directing the delivery of medical care and assisting patients in accessing health care services, patient care coordinators are often a point of contact among clinicians, patients, and family members.

### Interview Guide Development

The goal of our interviews was to identify potentially modifiable barriers and facilitators that cannot be gleaned from EHR but that if addressed may be associated with improved follow-up colonoscopy completion. The semistructured interview guide was developed through several iterations with the study team and was informed by social cognitive theory (Figure),^18^ which we selected for 3 reasons: (1) it is consistent with the social-ecological perspective that the health and behavior of individuals are determined by factors at multiple levels ranging from the intrapersonal to the societal; (2) it is a widely used theoretical framework in public health because it gives explicit guidance about methods for intervention development that promote health-enhancing behavioral change;^19^ and (3) it has been successfully applied to develop interventions to address a wide variety of health conditions and behaviors.^20^ The interviews took a mean of 30 to 40 minutes to complete.

**Figure.**
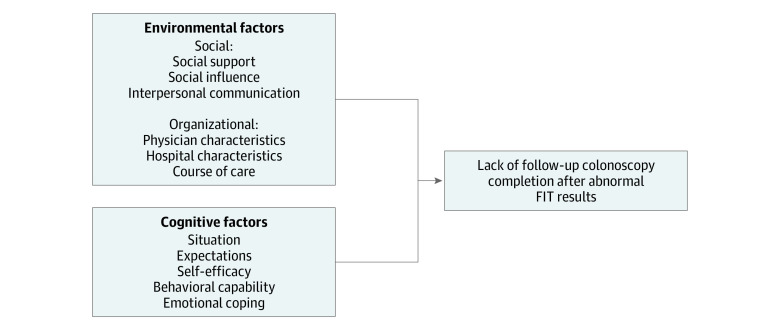
Conceptual Model for Barriers and Facilitators to Colonoscopy Completion FIT indicates fecal immunochemical test.

### Data Collection

Interviews were conducted and recorded by 3 authors (R.B.I., A.B.B., and D.A.) in person or via a secure conferencing platform from February to December 2020. All participants were assigned participant numbers and deidentified to the remaining research team. Interviews were transcribed verbatim, verified against recordings, and uploaded to data management software by participant number. Following accepted standards of rigor in qualitative research, we collected data until thematic saturation was achieved.^21^ To ensure our findings were relevant to diverse health care settings and clinicians, participants were asked to self-identify their race and ethnicity in an accompanying demographics survey.

### Statistical Analysis

Participant demographic data were described as proportions or medians and interquartile ranges (IQRs). For our qualitative analysis, we applied directed content analysis to all data.^22^ First, in a deductive approach, 3 authors (R.B.I., A.B.B., and C.S.), developed an initial list of codes and definitions that corresponded with research goals (eg, perceived barriers and facilitators to colonoscopy completion) and constructs of social cognitive theory (eg, social, organizational, and cognitive factors). Code labels and definitions were discussed to ensure accuracy. Next, in an inductive approach, coders independently reviewed a subset of the same interviews, created additional subcodes to reflect participants’ responses, and compared common themes and relevant quotes to ensure intercoder reliability. Finally, lead coders (R.B.I. and A.B.B.) independently applied the final codebook across interviews and extracted quotes illustrative of emergent themes for inclusion in the manuscript. Discrepancies were resolved through discussion and consensus between lead coders. We used Dedoose qualitative coding software version 8.3.35 2020 (SocioCultural Research Consultants) for data management.

## Results

Among 21 participants from HMC, 10 PCPs and 11 staff members were interviewed, and 20 participants (95.2%) provided demographic information. The median (IQR) participant age was 38.5 (33.0-51.5) years, and 17 (85.0%) were women. There were 10 participants (50.0%) who self-identified as Asian American or Pacific Islander, 7 participants (35.0%) who self-identified as White, and 2 participants (10.0%) who self-identified as Black or African American; 1 participant self-identified as Hispanic (5.0%), and 19 participants self-identified as non-Hispanic (95.0%). Among participants, 9 individuals (45.0%) spent more than 75% of their time providing patient care, and the median (IQR) time in practice was 8 (4-24) years (Table 1). Our qualitative content analysis found that barriers and facilitators to colonoscopy completion fell into 3 major themes: (1) environment: social determinants of health, (2) environment: organizational factors, and (3) cognitive factors, including but not limited to patient expectations, patient self-efficacy, and behavioral capacity as described to clinicians. Several subthemes were also identified and are summarized in the following sections. Representative quotes for clinician-identified barriers, facilitators, and potential solutions are summarized in Table 2.

**Table 1.** Interview Participant Characteristics

Characteristic	Participants, No. (%)		
	Total (N = 20)^a^	Physicians (n = 9)	Staff members (n = 11)^b^
Age, median (IQR)	38.5 (33.0-51.5)	35.0 (33.0-51.0)	42.0 (33.5-47.5)
Sex			
Men	3 (15.0)	2 (22.2)	1 (9.1)
Women	17 (85.0)	7 (77.8)	10 (90.9)
Race			
White	7 (35.0)	6 (66.7)	1 (9.1)
Black or African American	2 (10.0)	0	2 (18.2)
Asian American or Pacific Islander	10 (50.0)	2 (22.2)	8 (72.7)
Multiple or other	1 (5.0)	1 (11.1)	0
Ethnicity			
Non-Hispanic	19 (95.0)	8 (88.9)	11 (100)
Hispanic	1 (5.0)	1 (11.1)	0
Time practice, median (IQR), y	8.0 (4.0-24.0)	8.0 (4.0-24.0)	NA
Clinical effort, %^c^			
<20	2 (10.0)	0	2 (10.0)
20-50	6 (30.0)	3 (33.3)	3 (30.0)
50-75	3 (15.0)	2 (22.2)	1 (10.0)
>75	9 (45.0)	4 (44.4)	5 (50.0)

Abbreviations: IQR, interquartile range; NA, not applicable.

^a^ Among 21 interview participants, 1 physician did not complete the survey.

^b^ Staff members consisted of 3 patient care coordinators, 2 medical assistants, 2 registered nurses, 2 clinic managers, 1 physician assistant, and 1 caseworker.

^c^ Clinical effort is the proportion of participant’s time spent providing patient care.

**Table 2.** Key Themes, Subthemes, Supporting Quotations, and Potential Solutions

Theme and subthemes	Participants, No. (%) (N = 21)	Supporting quotation	Potential solution
**Barriers to follow-up colonoscopy**			
Environment: social determinants of health			
Lack of transportation	12 (57.1)	“There’s the transportation issue, people are often very reliant on Hopelink or a neighbor in this age range, or buses, etc, so that if it’s scheduled early, they’re late. If they’re late, they would get rescheduled, and they can’t keep doing that. They just don’t have the resources to keep being rescheduled for something they don’t really want anyhow.” — Physician	Explore nonconventional transportation options (eg, rideshares for colonoscopy completion)
		“I overheard another provider saying—this was not my patient—but who said that their patient found someone and just paid them money to come with him. Someone he didn’t know, which is really not great. He just found a random person on the street and gave them $40.” — Staff member	
Language barriers	11 (52.4)	“I know having 35 to 40 percent of patients who require an interpreter who may not even read in their preferred language, not gonna be able to read English, or understand all the intricacies of screening, I’m just a little bit worried about that I guess.” — Physician	Education materials in multiple languages Mastery training for interpreters specific to gastroenterology practice Education materials in pictorials
Homelessness	8 (38.1)	“And then, people who are marginally housed or experiencing homelessness who can’t quite do the prep very easily, particularly during this pandemic, when there’s not many facilities around to be able to do a prep. We’re not even ordering them right now because there’s no open access bathrooms for the most part, for people who are homeless.” — Physician	Government-sponsored affordable housing Increase in-hospital respite spaces
Environment: organizational factors			
Lack of care coordination	6 (28.6)	“I think the biggest barriers that our patients face would be coordination of care. A lot of the time, it is sort of getting them scheduled, getting them to complete the prep, to complete the prep correctly, and then coordinating a ride.” — Physician	Standardized protocols for abnormal FIT result tracking and follow-up EHR solutions to improve cross-specialty communication
Staffing shortages	4 (19.0)	“But basically, there are multiple times where I’m thinking, ‘Hey, this person has had whatever, a mammogram or a colonoscopy or an MRI, something ordered and there’s no movement on it.’ So, really, I feel like there’s a little bit of a burden on the provider to continue to follow up and make sure it actually happens more often than not I feel like. And so what I tend to end up having to do is reminding the patient care coordinators, I’m not trying to blame them, I think it’s a system issue. I think they probably swamped.” — Physician	Health system population health management Standardized protocols for abnormal FIT result tracking and follow-up EHR solutions to improve cross-specialty communication
COVID-19 pandemic	2 (9.5)	“And, then of course she has to do the COVID test the day before or two days before the test. And she’s just like in tears once she came to me, and said, ‘Can you call and cancel for me?’” — Staff member	Standardized protocols for abnormal FIT result tracking and follow-up EHR solutions to improve cross-specialty communication
Cognitive factors			
Challenges with bowel preparation	13 (61.9)	“We find that preparation has been really challenging for a lot of folks. Certainly, if they don’t have access to an individual bathroom, that’s really challenging. But even if we set up respite, or even if it’s at, say, someone who’s housed who has access, I think the conclusion of the prep is really challenging and could be due to health literacy.” — Physician	Government-sponsored affordable housing Increase in-hospital respite spaces Education materials in multiple languages Education materials in pictorials
Health literacy	10 (47.6)	“I also think another thing that can be huge is just the understanding of how important getting this screening is. If you have positive stool tests, and why it is so important, and you can talk to people about it, but also there is a huge range of health literacy in our clinic.” — Staff member	Education materials in multiple languages Education materials in pictorials Mastery training for interpreters specific to gastroenterology practice
Fear of procedure or cancer	9 (42.9)	“The third is just they’re just too afraid. They’re aware that they had a positive stool test, the doctors explained it to them but they’re still kind of ’I don’t know if I want to have this done. It’s my first one and my doctor said I needed it to get done’, and they just hold off on it.” — Staff member	Proactively discuss potential need for follow-up colonoscopy and patient fear
**Facilitators to follow-up colonoscopy**			
Environment: social determinants of health			
Interpretation services	10 (47.6)	“Now, we rely on people that are blocked interpreters that have done this a great deal, and so they’ll explain things. The doctors too will explain it, but it then gets reinforced. Because the patient always has questions, like, ‘Where’s this gonna happen?’ and ‘What did you say again?’ and it’s then going back to the provider, the trained interpreter will often just take over the conversation and explain it again.” — Physician	Mastery training for interpreters specific to gastroenterology practice
Insurance assistance	6 (28.6)	“We have a dedicated financial counselor at XYZ Clinic. So, when she is there, I would say I’m just really lucky, because I always had really, really good customer service and she’s been super helpful for individuals ... she will give me detailed instructions on what I need to do to follow up. So, it makes it a lot easier. I’m not trying to second guess what I need to be doing next.” — Staff member	Financial navigators
Transportation assistance	5 (23.8)	“We developed a relationship with a volunteer ride service; they’re called Sound Generations. Those are for patients who are 60 and above. They have a list of questions that we ask because they’re funded but they go out, pick up the patient, they’ll drop them off here. Once they’re done, they’ll pick them up and bring them home.” — Staff member	Explore nonconventional transportation options (eg, rideshares for colonoscopy completion)
Environment: organizational factors			
Patient care coordinators	14 (66.7)	“Yeah. I think the patient care coordinators are important in facilitating the process 100%. We couldn’t do it without them.” — Physician	Health system population health management Standardized protocols for abnormal FIT result tracking and follow-up EHR solutions to improve cross-specialty communication
Coordination across clinics	12 (57.1)	“I think the only one, that works really well is the actual coordination and actually if you have the patient on the line with you, because I feel like, you know, if you give the patient the number, or information for them to call and schedule, I feel like that kind of—because I feel like there’s so much hesitation in a patient’s perspective scheduling these procedures, not to mention, especially from my background giving GI procedures, there’s so much, socio-cultural stuff that can really prevent the patient, or having them think twice before they even call and schedule, if that makes sense.” — Staff member	Health system population health management Standardized protocols for abnormal FIT result tracking and follow-up EHR solutions to improve cross-specialty communication
		“I rarely have them schedule it on their own. It’s just that access to the telephone is limited here and people usually only have a cell phone and they’re limited to the number of minutes they may have, so it’s better for me to make the appointment because it can be challenging unless you get directly to a patient care coordinator. It makes a big difference.” — Staff member	
Appointment reminders	7 (33.3)	“Yeah, so after we schedule (the colonoscopy) we send them a letter and then our medical assistants actually call them seven days and three days (before the procedure).” — Staff member	Health system population health management Standardized protocols for abnormal FIT result tracking and follow-up EHR solutions to improve cross-specialty communication
Cognitive factors			
In-person follow-up	10 (47.6)	“Like for some of our patients, honestly we have to bring them back for another visit to even discuss this abnormal result because of either health literacy or language barriers and so, sometimes we do that.” — Physician	Group in-person or virtual education sessions Peer support and education networks
General patient education	7 (33.3)	“I always, even before we would order that, explain that if we do a FIT test, there is a possibility that if it’s abnormal, we have to do a colonoscopy, so I explain that every time.” — Physician	Proactively discuss potential need for follow-up colonoscopy and patient fear Peer support and education networks
Bowel preparation education	2 (9.5)	“So, I think in-person counseling for prep has worked well when our nurses can do it or when I can do it and when patients can show. And then arranging respite and a place for preparation for folks who don’t otherwise have a place for preparation.” — Physician	Group in-person or virtual education sessions Peer support and education networks

Abbreviations: EHR, electronic health record; FIT, fecal immunochemical test.

### Barriers to Colonoscopy Completion

#### Environment

##### Social Determinants of Health

Topics related to social determinants of health among patients were the most common barriers to colonoscopy completion identified by interview participants. Lack of patient transportation (12 participants [57.1%]), language barriers (11 participants [52.4%]), and homelessness (8 participants [38.1%]) were among the most frequently reported factors. Participants remarked that patients frequently lacked access to individuals who could accompany them or drive them to and from the endoscopy unit. A substantial proportion of patients required an interpreter, and study participants were concerned about the accuracy of interpretations given the intricacies of colonoscopy completion. For patients experiencing homelessness, participants noted that the lack of access to telephones for colonoscopy instructions and restrooms to complete bowel preparation were significant challenges to colonoscopy completion.

##### Organizational Factors

The most frequently reported organizational barriers were lack of care coordination between primary and specialty care clinics (6 participants [28.6%]), staffing shortages (4 participants [19.0%]), and the COVID-19 pandemic (2 participants [9.5%]). Participants highlighted that clinical and regulatory procedures (eg, gastroenterology referral reviews and insurance approvals) were associated with interrupted communications between primary and specialty care and barriers to colonoscopy completion. Participants also noted that inconsistent workflows for folllow-up of abnormal FIT results across the health system, multiple steps in colonoscopy coordination, and employee turnover owing to the academic environment were associated with delayed colonoscopy completion.

#### Cognitive Factors

Physicians and staff members reported patient cognitive factors as a common barrier to colonoscopy completion. These were most commonly associated with patient challenges with procedure bowel preparation (13 participants [61.9%]), limited health literacy (10 participants [47.6%]), and fear of the procedure or a cancer diagnosis (9 participants [42.9%]). Participants stated that among older patients and those with impaired mobility or multiple medical problems, lack of confidence in their ability to complete bowel preparation was a significant barrier to colonoscopy completion. Participants also expressed that many patients did not understand the significance of an abnormal FIT result or the importance of a follow-up colonoscopy.

### Facilitators to Colonoscopy Completion

#### Environment

##### Social Determinants of Health

When participants were asked about facilitators that addressed social determinants of health barriers, interpretation services (10 participants [47.6%]), insurance assistance (6 participants [28.6%]), and transportation assistance (5 participants [23.8%]) were most frequently reported. In 1 clinic, a participant noted that having interpreters who were more familiar with gastrointestinal procedures was associated with improved patient knowledge and colonoscopy completion. Another participant highlighted that having a dedicated financial counselor in the clinic was associated with streamlined colonoscopy referrals and procedures.

##### Organizational Factors

The most frequently reported organizational facilitators included having adequate staffing, specifically patient care coordinators (14 participants [66.7%]); care coordination across primary and specialty care clinics (12 participants [57.1%]); and patient appointment reminders (7 participants [33.3%]). Participants shared that during periods of adequate staffing, patient care coordinators performed patient navigation activities, including assisting with scheduling CRC screening tests and follow-up of abnormal screening results. Interview participants who were patient care coordinators also discussed how they leveraged health system knowledge and relationships to assist patients. For example, they connected patients with social workers to assist with logistical barriers, including access to health insurance and transportation.

#### Cognitive Factors

The most frequently reported facilitators that addressed cognitive barriers to colonoscopy completion were scheduling in-person follow-up appointments (10 participants [47.6%]), general patient education (7 participants [33.3%]), and specific bowel preparation education (2 participants [9.5%]). Participants stated that setting patient expectations concerning FIT-based CRC screening (eg, informing patients about the 2-step nature of stool-based CRC screening) was associated with improved follow-up of abnormal FIT results. Participants also noted that for patients who were experiencing homelessness or whose primary language was not English, scheduling an in-person visit to review FIT results and introducing bowel prep instructions were associated with improved ability among staff members to schedule follow-up colonoscopy appointments during those visits and patient empowerment to complete the colonoscopy.

## Discussion

To determine clinician-identified barriers and facilitators to colonoscopy completion among patients in a safety net health care system undergoing CRC screening with an abnormal FIT result, we conducted a qualitative study using semistructured interviews of PCPs and staff members. Our analysis found that barriers to follow-up colonoscopy completion included social determinants of health (eg, lack of patient transportation, language barriers, and homelessness), organizational factors (eg, lack of care coordination, staffing shortages, and COVID-19–related practice changes), and patient cognitive factors (eg, challenges with bowel preparation, health literacy, and fear of colonoscopies). Our analysis also found that existing resources that addressed these barriers (eg, insurance assistance, appointment reminders, and bowel preparation education) were associated with improved colonoscopy completion but were insufficient to meet the recommended follow-up colonoscopy goal of 80%.^23^ The strengths of this study are its diverse participant population, whose practice setting fills an important void in the existing literature, and the qualitative study design, which allowed for detailed inquiry not otherwise possible through EHR review.

Colonoscopy completion is a complex process that may be especially challenging for patients in safety net health systems who experience fragmented care. Owing to socioeconomic factors, patients in safety net health care systems are less likely to receive follow-up specialty care compared with patients in higher-income groups.^24^ Through EHR analysis, we previously reported that lack of clinician referral, complex medical and social issues, and missed colonoscopy appointments after scheduling were associated with a lack of colonoscopy completion after abnormal FIT results in a safety net health system.^10^ While 2 studies from 2021^13,14^ signaled that patient navigation may be associated with improved colonoscopy completion, qualitative studies of clinician-identified barriers that may inform interventions to further improve follow-up colonoscopy completion are limited.^25,26,27^ Through semistructured clinician interviews, our study extends the existing literature by identifying potential areas of intervention within safety net health systems at the patient level (eg, patient transportation), clinician level (eg, patient education), and health system level (eg, primary and specialty care coordination).

From 2010 to 2016, Kaiser Permanente Northern California, an integrated managed care organization, implemented several strategies to improve follow-up colonoscopy completion. Over 10 years, the organization hired additional gastroenterology personnel to expand endoscopy capacity, created a central registry of abnormal FIT results, standardized outreach through patient navigation, designated an individual responsible for tracking patients with abnormal FIT results, assigned follow-up of abnormal FIT results to gastroenterology departments, mailed certified letters to patients who did not respond to navigators, and adopted a quality metric to achieve 80% follow-up colonoscopy completion within 30 days of an abnormal FIT. These combined strategies were associated with an increase in follow-up colonoscopy completion from 73% to 85%.^28^ While it may be challenging for safety net health systems and FQHCs to replicate these efforts, our study suggests potential patient-level, clinician-level, and system-level strategies that may be adopted in resourced health care settings with lower resource levels that frequently use FIT for CRC screening.

Inadequate follow-up colonoscopy has been well described in safety net health systems, but few interventions have been evaluated for these settings. In the Los Angeles County Department of Health Services, an integrated safety net system, implementation of a patient navigation program was associated with an increase in colonoscopy completion after abnormal FIT results by 5.4% (40.6% to 46%).^14^ In the San Francisco Health Network, an integrated safety net system, we identified 3 trends in clinics with increased rates of follow-up colonoscopy completion 1-year after an abnormal FIT result: (1) Higher-performing clinics used registries to track patients with abnormal FIT results until colonoscopy completion. (2) Higher-performing clinics assigned at least 2 team members to communicate abnormal FIT results to patients. (3) Team members responsible for communicating FIT results consistently included a nurse and medical assistant.^29^ Our study adds qualitative perspectives from PCPs and staff members to refine interventions in safety net health care systems and similar settings with limited resources and may assist health systems in prioritizing resources to support colonoscopy completion in these settings.

Our study highlights several potential opportunities to improve follow-up colonoscopy completion among patients in safety net health care systems with abnormal FIT results (Table 2). For example, rideshare interventions have been explored to address transportation barriers in primary care settings with mixed results,^30,31^ but rideshare interventions have not yet been explored for colonoscopy completion due to associated procedural sedation. Optimizing rideshare use for colonoscopy may address transportation barriers identified in this study. Additionally, as health systems explore value-based models of care, this creates opportunities to increase patient care coordination through population health management to improve follow-up colonoscopy completion. For example, having gastroenterology, rather than primary care, directly follow up on abnormal stool-based screening test results has been associated with decreased colonoscopy wait times.^32^ Such a model could improve familiarity and communication between patient care coordinators in primary care and gastroenterology clinics and improve follow-up colonoscopy completion.^33^

### Limitations

This study has several limitations. First, we interviewed a subset of key informants who were involved in the care of patients with abnormal FIT results. Given that qualitative studies often rely on smaller samples than quantitative studies, it is possible that the barriers and facilitators identified by participants may differ from what those who were not interviewed would identify. Second, our study was conducted in an urban safety net county teaching hospital, and given this unique patient population, our findings may not be generalizable to different primary care settings. Third, while patient interviews to complement our findings are ongoing, we did not conduct clinician-patient dyad interviews, and it is possible that in dyad conversations, patients would have identified different barriers and facilitators than their clinicians did.

## Conclusions

In this qualitative study of safety net health system PCPs and staff members, we identified barriers to follow-up colonoscopy completion, including environmental factors (ie, social determinants of health and organizational factors) and patient cognitive factors. Participants stated that resources that addressed these barriers (eg, insurance assistance, appointment reminders, and bowel preparation education) were associated with improved colonoscopy completion, but the facilitators identified have yet to be systematically implemented or evaluated. Incorporating clinician-identified factors into multilevel interventions may be associated with improved colonoscopy completion among patients with abnormal FIT results and help address one of the most persistent challenges in cancer prevention and control for safety net and other medically underserved populations.

## Notes

Supplement. **eTable.** Health Services Research Terms and Definitions
eReferences.
