# Supplementary material for: Perceptions on Barriers and Facilitators to Colonoscopy Completion After Abnormal Fecal Immunochemical Test Results in a Safety Net System
Source: JAMA Netw Open. 2021 Aug 10;4(8):e2120159. doi: 10.1001/jamanetworkopen.2021.20159 (PMC8356069; doi:10.1001/jamanetworkopen.2021.20159)
Supplement: Supplement. — eTable. Health Services Research Terms and Definitions eReferences. [file jamanetwopen-e2120159-s001.pdf]

Supplemental Online Content

Issaka RB, Bell-Brown A, Snyder C, et al. Perceptions on barriers and facilitators to colonoscopy completion after abnormal fecal immunochemical test results in a safety net system. *JAMA Netw Open*. 2021;4(8):e2120159. doi:10.1001/jamanetworkopen.2021.20159

eTable. Health Services Research Terms and Definitions

eReferences

This supplemental material has been provided by the authors to give readers additional information about their work.

© 2021 Issaka RB et al. *JAMA Network Open.*

eTable. Health Services Research Terms and Definitions

Term	Definition
Safety-net system	Providers that organize and deliver a significant level of health care and other needed services to uninsured, Medicaid and other vulnerable patients.[1]
Federally Qualified Health Centers (FQHCs)	Community-based health care providers that receive funds from the Health Resources & Services Administration (HRSA) Health Center Program to provide primary care services in underserved areas. They must meet requirements such as providing care on a sliding fee scale and operating under a governing board that includes patients.[2]
Social Cognitive Theory	Describes the influence of individual experiences, the actions of others, and environmental factors on individual health behaviors.[3]
Social Determinants of Health	The conditions in the environments where people live and operate that affect health, functioning, and quality-of-life outcomes and risks.[4]
Socioeconomic status	The social standing or class of an individual or group. Often measured as a combination of education, income, and occupation.[5]

eReferences

1. Lewin ME, Altman S, eds. America's health care safety net: intact but endangered. Washington, DC: National Academy Press; 2000. Available at: http://books.nap.edu/catalog/9612.html

2. Health Resources & Services Administration. Federally Qualified Health Centers. https://www.hrsa.gov/opa/eligibility-and-registration/health-centers/fqhc/index.html (2018, accessed May 19, 2021).

3. Rural Health Information Hub. Social Cognitive Theory. https://www.ruralhealthinfo.org/toolkits/health-promotion/2/theories-and-models/social-cognitive (2018, accessed May 19, 2021).

4. US Department of Health and Human Services, Office of Disease Prevention and Health Promotion. Healthy People 2030, Social Determinants of Health. https://health.gov/healthypeople/objectives-and-data/social-determinants-health (Accessed May 20, 2021).

5. American Psychological Association. Socioeconomic Status. https://www.apa.org/topics/socioeconomic-status (Accessed May 20, 2021).

© 2021 Issaka RB et al. *JAMA Network Open.*
